# Klebsiella sp.-related infectious spondylitis in a bearded dragon (*Pogona vitticeps*)

**DOI:** 10.1186/s12917-021-02933-7

**Published:** 2021-06-29

**Authors:** Alessandro Vetere, Mara Bertocchi, Igor Pelizzone, Emanuele Moggia, Sebastiano Gerosa, Francesco Di Ianni

**Affiliations:** 1 Clinica Veterinaria Modena Sud, Piazza dei Tintori, 1, Spilamberto (MO), Italy; 2 Department of Veterinary Science, University of Parma, Strada del Taglio 10, 43126 Parma (PR), Italy; 3 Ambulatorio Veterinario Belvedere, Via Pietro Bembo 12, 42123 Reggio Emilia (RE), Italy; 4 Ambulatorio Veterinario Levante, Via Alberto Salietti, 6, 16043, Chiavari (GE), Italy; 5 Ospedale Veterinario San Francesco, via Newton 2, 20148 Milano (MI), Italy

**Keywords:** Spondylitis, *Klebsiella* sp, *Pogona vitticeps*, Pet lizard, Reptiles

## Abstract

**Background:**

Spondylitis is an inflammation of the vertebrae that leads to a destructive process with exuberant new bone formation. Osteomyelitis can produce a distortion of the bone architecture, degenerative joint changes and ankyloses of adjacent vertebrae. In reptiles, intervertebral discs are absent, so the term discospondylitis is not used. In lizards, vertebral lesions have not been well studied. The present paper describes the first case of *Klebsiella* sp.-related spondylitis in a pet lizard (*Pogona vitticeps*).

**Case presentation:**

A 2-year-old, female bearded dragon (*Pogona vitticeps*) was presented for clinical examination due to a decreased activity level, decreased appetite and constipation. Blood tests showed no remarkable alterations. The haemogram showed normal parameters with relative lymphocytosis, although the absolute number of lymphocytes did not differ from the reference values. A computed tomography scan revealed a mixed osteolytic-proliferative bone lesion diffusing to the first and last tracts of the pre-sacral vertebrae together. A small amount of material obtained from the spinal swelling was sampled with an aseptic technique for bacterial culture, which was positive for *Klebsiella* sp*.* The antibiogram revealed sensitivity to enrofloxacin, marbofloxacin, and chloramphenicol and intermediate sensitivity to gentamicin. Complete return to spontaneous feeding was achieved 15 days after the beginning of antibiotic and anti-inflammatory therapy.

**Conclusions:**

In veterinary medicine, spondylitis represents a well-known disease in small companion animals. In mammals, the most common aetiologic agents are fungi and bacteria. Antibiotic therapy was set based on the antibiogram, and marbofloxacin was chosen at a dosage of 10 mg/kg subcutaneously (SC) once per day (SID). After only 7 days of antibiotic therapy, the clinical condition improved significantly; the patient started feeding and drinking spontaneously and gained weight. This case should remind clinicians of the importance of always performing antibiograms before choosing any antibiotic therapy. Considering reptiles, there have been few papers about spinal diseases, mostly regarding snakes and a few about *Iguana iguana*. Relative to other species of saurians, the literature remains lacking.

## Background

Spondylitis is inflammation of the vertebrae that leads to a destructive process with exuberant new bone formation [1]. Osteomyelitis can produce a distortion of the bone architecture, degenerative joint changes and ankyloses of adjacent vertebrae [2]. In reptiles, intervertebral discs are absent [1], so the term discospondylitis is not used. In snakes, proliferative osteoarthritis and osteoarthrosis have been reported, and three histologic types of lesions have been found: active bacterial osteoarthritis, predominantly noninflammatory osteoarthrosis with multifocal inflammation suggestive of chronic bacterial osteoarthritis, and noninflammatory lesions consistent with osteoarthrosis without evidence of inflammation or bacteria. These findings suggest that all of these snakes presented a single disease process: bacterial infection of the vertebrae. The different histologic lesions observed in these snakes could be a continuum of lesions, from acute to chronic. The bacteria involved were Salmonella spp. and Streptococcus spp. [2, 3]. In lizards, vertebral lesions have not been well studied, and the pathogenesis of these lesions requires further study. *Klebsiella* spp. are Gram-negative, rod-shaped bacteria that belong to the family *Enterobacteriaceae,* which are responsible for fermenting lactose. As with the other coliform lactose fermenters, *Klebsiella* spp. are facultative anaerobic and oxidase-negative bacteria. The genus *Klebsiella* contains nonmotile encapsulated organisms that hydrolyse urea, ferment inositol and utilize citrate. Species of veterinary interest are *K. pneumoniae* and *K. oxytoca*. *Klebsiella* spp. are ubiquitous in nature, where they are found in surface water, sewage, soil and plant material [4]. They have also been isolated from the gastrointestinal tract of healthy mammals and reptiles [4, 5]. Although *Klebsiella* spp. are considered normal flora by some clinicians; when they are isolated in pure culture or from clinically ill reptiles, the patient should be treated [6] because they could be responsible for different infections, including stomatitis and enteritis [5]. *Klebsiella* spp. are opportunistic pathogens. Virulence factors can include capsules, endotoxins, enterotoxins, adhesins, and siderophores. The capsule is essential to virulence; it has antiphagocytic properties and prevents killing by bactericidal factors in serum. Endotoxins are responsible for fever, neutropenia, petechiae and ecchymosis, shock, pulmonary oedema and vascular collapse in coliform septicaemia [4]. *Klebsiella pneumoniae* ssp*. pneumoniae* has been associated with several infectious processes in animals, particularly bovine mastitis, equine metritis, joint illness and neonatal septicaemia in foals and calves, and different infections in dogs, pet birds, poultry and captive reptiles [4]. In the latter species, this bacterium has also been isolated from various individuals with pneumonia and hypopyon and from snakes and iguanids with osteomyelitis [4]. Classes of antibiotics suggested for treating *Klebsiella* spp. are aminoglycosides and cephalosporins [6]. Aminoglycosides are considered the gold standard for Gram-negative sepsis and are generally indicated for Gram-negative bacterial infections [4]. To treat *Klebsiella* spp., marbofloxacin could also be used. Marbofloxacin is a synthetic, broad-spectrum, bactericidal fluoroquinolone antibiotic that acts to inhibit DNA gyrase [7]. According to the literature, the recommended dose is 10 mg/kg PO every 48 h in snakes [8] and 2 mg/kg IV or IM every 24 h in chelonians [9]. Exotic animals are becoming increasingly common pets. It is therefore essential to rely on expert practitioners, as well as to deepen the knowledge related to the diagnostic and therapeutic paths for these species [10–12].

## Case presentation

**Fig. 1:**
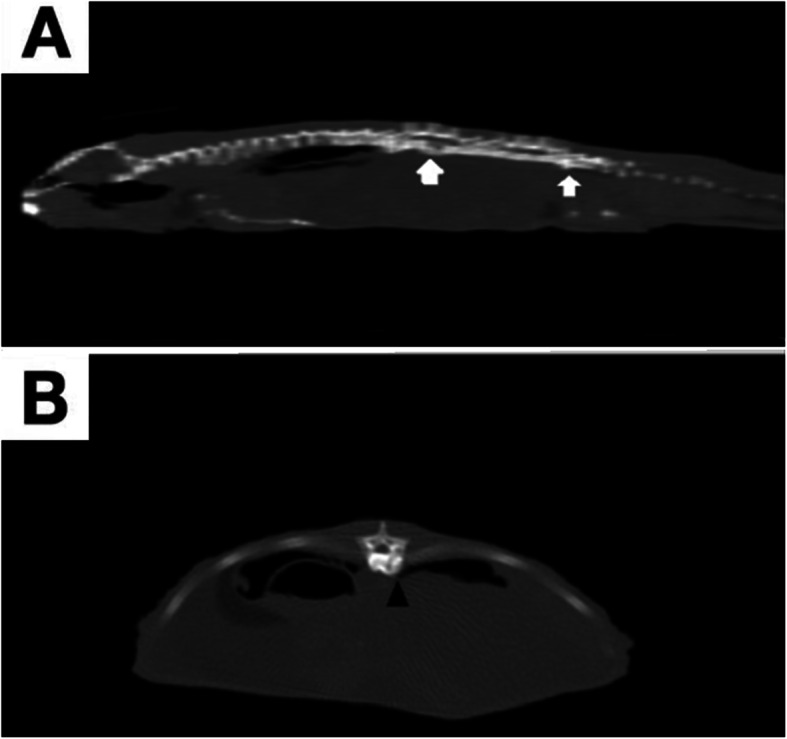
**A**: The CT scan revealed a mixed osteolytic-proliferative bone lesion diffusing to the first and last tracts of the pre-sacral vertebrae together (white arrows). **B**: An equal combination of the bone destructive process and new bone production was evident in all lesions (black arrowhead, axial plane)

A 2-year-old, 200-g female bearded dragon (*Pogona vitticeps*) was presented for clinical examination due to decreased activity levels, decreased appetite and constipation. The body condition score was 4 of 5, and minimal dehydration (<2.5%) was achieved. The patient also showed two mild swellings on its back: the first was approximately at the last tract of the pre-sacral vertebrae, and the second was localized at the first third of the post-sacral vertebrae. The animal was housed in a glass terrarium at 30 °C (86 °F) at day and 25 °C (77 °F) at night. The diet was balanced with insects dusted with calcium powder twice per week alternating with fresh vegetables weekly. A UVB light 5.0 spectrum was provided and changed every 6 months. Complete blood work, X-rays and a CT scan were performed. Blood tests showed no remarkable alterations (Table 1) [13, 14]. The haemogram showed normal parameters with relative lymphocytosis, although the absolute number of lymphocytes did not differ from the reference values. X-rays showed appreciable alterations only in LL projection, and mild to moderate osteolysis of the first and last tracts of the pre-sacral vertebrae was evident. Radiograms did not show significant alterations of coelomatic organs. However, a total body 2.5-mm interval CT scan without contrast medium administration was also performed to evaluate the spinal bone condition. The CT scan revealed a mixed osteolytic-proliferative bone lesion diffusing to the first and last tracts of the pre-sacral vertebrae together (Fig. 1). Equal combinations of bone destructive processes and new bone production were evident. Cytology of the swelling was performed, but the cellularity was not diagnostic. A small amount of material obtained from spinal swelling was used for a bacterial culture, which was positive for *Klebsiella* sp*.* The antibiogram revealed sensitivity to enrofloxacin, marbofloxacin, and chloramphenicol and intermediate sensitivity to gentamicin. The patient was discharged after 1 week of assisted feeding with crickets and wax worms and oral rehydration with ringer solution (S.a.l.f. Spa, Via Guglielmo Marconi, 2, 24069 Cenate sotto BG (Italy)) with an indication for daily administration of meloxicam (Ceva Salute Animale S.p. A, Viale Bartolomeo Colleoni, 15, 20864 Agrate Brianza (MB), Italy) at a dosage of 0.2 mg/kg once per day (SID) SC for 1 week and marbofloxacin (20 mg/ml) (Marbocyl® 2%, *Vétoquinol Italia* S.r.l., Via Piana, 265 - 47032 Bertinoro (FC) Italy) at 10 mg/kg [15] SID SC for 2 weeks. Seven days after starting therapy, the owner reported clinical improvement, weight gain (212 g) and reprise of spontaneous feeding and drinking. Complete return to spontaneous feeding was achieved 15 days after the beginning of antibiotic and anti-inflammatory therapy. At the 30-day follow-up visit, spinal swelling was indeed present, but a clinical improvement was noted. No alterations in CBC were found.

**Table 1:** Comparison between biochemical values and CBC (Complete Blood Count) parameters of the patient and the reference values

Analyte	Case	Reference Values
Hct (%)	26	17–45^a^
RBC (10^6^/μL)	0.8	0.40–1.60^a^
Hgb (g/dL)	7.5	4.7–14^a^
WBC (10^3^/μL)	5.5	1.45–19.0^a^
Heterophils (10^3^/μL)	0.33	0.24–7.77^a^
Lymphocytes (10^3^/μL)	5.06	0.29–11.3^a^
Monocytes (10^3^/μL)	0.11	0.03–1.39^a^
Azurophils (10^3^/μL)	0	0.01–1.98^a^
Eosinophils (10^3^/μL)	0	0.01–1.37^a^
Basophils (10^3^/μL)	0	0.04–1.28 ^a^
Uric Acid (mg/dL)	3.74	0.5–9.8^b^
Calcium (mg/dL)	2.8	2.15–4.49^b^
Phosphorus (mmol/L)	1.2	0.68–3.42^b^
ALT (U/L)	10	0–33^b^
AST (U/L)	43	2–90^b^
Glucose (mg/dL)	225	108–333^b^
Urea (mg/dL)	1	–
Protein, total (g/L)	33	12–40^b^
Albumin (g/L)	15	–
LDH (U/L)	285	25–1906^b^
Creatine kinase (U/L)	2475	33–4042^b^

^a^ [10]

^b^ [11]

## Discussion and conclusions

In veterinary medicine, spondylitis represents a well-known disease in small companion animals [16, 17], equines [18] and birds [19]. In reptiles, there have been few papers about spinal diseases, most regarding snakes [3] and few about iguana species (*Iguana iguana*) [2]. In other species of saurians, the literature has been lacking. In mammals, the most common aetiologic agents are fungi [20], such as *Aspergillus terreus,* and bacteria, such as *Pseudomonas aeruginosa, Enterococcus faecalis* and *Staphylococcus epidermidis* [21, 22]. The pathophysiology of spondylitis is not completely clear. One theory is that the presence of subchondral vascular loops in the vertebral epiphysis slows circulation, allowing for colonization of blood-borne bacteria, which then diffuse through the cartilaginous endplate of the vertebral body to reach the disc [21]. Infection is further disseminated to adjacent vertebrae by freely communicating venous sinuses [21]. Foreign bodies, including grass awns, have been associated with spondylitis in mammals [23]. In reptiles, pathogenesis has been poorly understood to date [2]. In squamates, physical examination does not always provide sufficient information. Diagnostic imaging techniques are useful tools for the examination of reptiles [24]. In particular, CT is a noninvasive, cross-sectional diagnostic imaging technique that offers significant advantages for the detection of pathologies in small animal practice and indeed in the exotic animal field [25]. CT is ideal for diagnosing skeletal diseases and pathologic processes involving soft tissues [24]. Changes on the haemogram in reptiles reflect the trend of pathophysiology in mammals: an increase in leukocytes, particularly lymphocytes, monocytes and heterophils, is associated with acute, subacute or chronic inflammation associated with infection, wound healing, parasitism, and viral disease [26]. Lymphocytes are the most common leukocytes, accounting for up to 80% of the differential healthiest reptiles, although exceptions to this general rule exist [26]. Antibiotic therapy was chosen based on the antibiogram, which confirmed the well-founded suspicion of an ongoing infection. Among the effective antibiotics, marbofloxacin was chosen due to the potential nephrotoxicity reported for gentamicin [15]. Chloramphenicol is considered a last resort antibiotic due to the risk of antibiotic resistance [15]. No alterations in clinical chemistry were found [14] despite the reference values (Table 1). *Klebsiella* spp. are opportunistic pathogens. *Klebsiella pneumoniae* is considered an opportunistic pathogen in humans, constituting an ongoing health concern for immunocompromised patients, the elderly, and neonates [27]. Over the past 30 years, interest in reptiles as pets has increased, and this interest can also be observed worldwide. In all cases, travel and new habitats exert stress on animals, favouring the faecal excretion of intestinal pathogens, such as Klebsiella spp. and Enterobacter spp., which are able to produce urinary tract infections and septicaemia in humans [28, 29]. This case report should raise awareness among reptile breeders to consider preventive measures, such as rigorous personal hygiene after contact with a newly introduced pet in the collection and the use of protective equipment, especially when handling animals showing clinical signs of disease, such as anorexia, vomiting or diarrhoea.

## Data Availability

All of the data generated or analysed during this study are included in this published article [and its supplementary information files].

## Abbreviations

°C: Degrees Celsius
°F: Degrees Fahrenheit
AST: Aspartate aminotransferase
BUN: Blood urea nitrogen
CBC: Complete blood count
CT: Computed tomography
Hct: Haematocrit
Hgb: Haemoglobin
IM: Intramuscular
IV: Intravenous
LDH: Lactic acid dehydrogenase
LL: Latero-lateral
MCH: Mean corpuscular haemoglobin
MCHC: Mean corpuscular haemoglobin concentration
MCV: Mean corpuscular volume
RBC: Red blood cell
SC: Subcutaneous
SID: Once daily
WBC: White blood cell
